# Liver Enzymes Changes and their Association with Outcome in Hospital Inpatients with COVID-19 in Jordan

**DOI:** 10.34172/mejdd.2025.414

**Published:** 2025-04-30

**Authors:** Tarek Mazzawi, Hadeel Alshahwan, Ban Aladamat, Sara Al.Nsour, Leen Asfour, Dana Abu Hanak, Seema Al-Shehab

**Affiliations:** ^1^Department of Internal Medicine, Faculty of Medicine, Al-Balqa Applied University, Salt, Jordan

**Keywords:** Hepatitis, Liver injury, Viral infection

## Abstract

**Background:**

Abnormal levels of liver enzymes have been reported in patients with COVID-19 and severe clinical presentation. However, limited studies exist in our region regarding the impact of COVID-19 on the liver’s function. Thus, we aimed to investigate liver enzyme changes and their association with prognosis and outcome in hospitalized patients with COVID-19 in Jordan.

**Methods:**

This retrospective cohort study included 359 patients with COVID-19 who were admitted to Ministry of Health hospitals all around Jordan during the second pandemic wave of COVID-19. Data such as liver enzymes, patients demographics, and outcomes were collected and statistically analysed.

**Results:**

Approximately 39.6% of infected patients had elevated liver enzymes, and 5.9% had elevated enzymes more than twice the upper limit of normal (ULN). Among these patients, 40.8% had both aspartate aminotransferases (AST) and alanine aminotransferases (ALT) elevation, 52.1% had AST elevation alone, and 7.0% had ALT elevation alone. Factors associated with worse prognosis and outcome were older age, male sex, and comorbid autoimmune conditions. The peak and at-discharge values of AST and ALT showed significant differences.

**Conclusion:**

Liver enzyme abnormality is common among patients with COVID-19 and AST is the most common abnormality. AST and ALT elevation can be associated with longer hospital stays, older age, and male sex.

## Introduction

 COVID-19 is an infectious disease caused by a new coronavirus called SARS-CoV-2. It joined the other two zoonotic coronavirus diseases, severe acute respiratory syndrome (SARS) and Middle East respiratory syndrome (MERS).^1^ The World Health Organization (WHO) announced the 2019‐nCoV epidemic as a public health emergency of international concern on the 30^th^ of January 2020.^2^ The outbreak has created numerous challenges around the world and impacted individuals from all populations and across all demographics. The number of COVID-19 cases in Jordan has seen a notable rise. Up to February 21, 2023 there have been 1 746 997 confirmed cases, with 14 122 deaths reported to the WHO. The infection is commonly manifested by fever, dry cough, breathing difficulties (dyspnea), headache, and pneumonia that may result in progressive respiratory failure and even death.^1^

 While COVID-19 mainly affects the pulmonary system causing respiratory disease (pneumonia and acute respiratory distress), there is some evidence that it has a multi-systemic nature leading to extrapulmonary manifestations such as hematological complications (like lymphopenia), myocardial dysfunction and arrhythmia, acute coronary syndromes, acute kidney injury, gastrointestinal symptoms mainly diarrhea, abdominal pain and vomiting, hyperglycemia and ketosis, neurological and ophthalmological manifestations such as headache, dizziness, conjunctivitis, and dermatological symptoms such as erythematous rash.^3^

 Moreover, a prior review has demonstrated that some patients with COVID-19 have hepatic injury manifested as alanine aminotransferases (ALT) and aspartate aminotransferases (AST) elevation that is commonly noticed in severe COVID-19 infections.^4^ Liver enzyme elevation can be considered a predictive factor for disease course as patients who have significantly elevated levels have higher odds of hospital admission, intensive care unit (ICU) admission, intubation, and death.^5^

 Several studies from China assessing the levels of liver enzymes in COVID-19 admitted patients found that most patients showed abnormalities in liver enzymes either during hospitalization,^6^ or 2-3 weeks after admission.^7^ Some studies from the USA found a correlation between abnormal liver enzymes and worse outcomes for COVID-19 patients, with the strongest association observed between peak liver enzymes and severe infection (defined as ICU admission, respiratory failure requiring mechanical ventilation, use of vasopressor therapy, or death).^8,9^ In Iraq, a study found that many patients with COVID-19 had liver enzyme abnormalities, with total bilirubin (BIL) followed by AST being the most elevated.^10^ In Iran, a study found a significant correlation between the severity of disease (such as the need for ICU admission) and elevated levels of AST and direct BIL, whereas the mortality rate was significantly increased with AST levels only.^11^ Multiple studies observed the association between the elevation of liver enzymes and the use of drugs during the in-hospital stay.^6,8,12^ Drugs such as antibiotics, non-steroidal anti-inflammatory drugs, ribavirin, herbal medications, and interferon did not increase the risk of liver injury, according to Cai and colleagues, whereas lopinavir/ritonavir did by four times.^6^ Moreover, Zhenyu Fan and others found similar results, with a higher prevalence of receiving treatment with lopinavir/ritonavir among patients with abnormal liver function than those with normal liver function.^12^ In addition, hospital inpatients with peak AST/ALT elevation were found to be associated with the medications: lopinavir/ritonavir, hydroxychloroquine, remdesivir, and tocilizumab.^8^

 Since there are conflicting results and limited studies regarding the impact of COVID-19 on liver enzymes, severity of disease, and length of stay, we aimed to investigate liver enzyme changes and association with prognosis and outcome in hospital inpatients with COVID-19 in Jordan.

## Materials and Methods

###  Study Type and Population

 The second (and more aggressive) wave of COVID-19 in Jordan started in January 2021, peaking during mid-March 2021 and lasting until May 2021.^13^ Therefore, we based our retrospective cohort study population on COVID-19-positive patients admitted to Ministry of Health hospitals around Jordan from March 1st to March 31, 2021, with 605 007 confirmed cases overall during that month, according to the WHO.

 The population size was 4233 in total, for which liver enzymes were assessed for only 528 patients. The inclusion criteria were patients above 18 years of age, at least one AST and ALT reading for patients with normal liver enzyme levels, and at least two ALT and AST readings for patients with elevated liver enzymes, the first being at admission, and the second being at another time-point during hospitalization or at discharge. Patients who are pregnant, with pre-existing liver disease, or incomplete medical records were excluded. This yielded 359 patients in our study, with 142 patients with liver enzyme abnormalities.

 The diagnosis of COVID-19 was confirmed by reverse transcription polymerase chain reaction (RT-PCR), and the patients’ medical records have been included from admission till the outcome of the disease course (discharge or death).

###  Data Collection Methods

 Data were collected using electronic patient records (Hakeem) of patients who were admitted to the Internal Medicine ward, COVID-19 ward, or ICU ward as confirmed cases of COVID-19.

 The collected data included demographics (age, sex), date of admission and discharge, comorbidities, COVID-19 symptoms including the presenting symptom, vital signs, the outcome of admission (death or discharge), the type of oxygen delivery device, need for ICU ward admission, and the following lab test results: routine laboratory inflammatory markers (c-reactive protein (CRP) and D-dimer), total BIL, international normalized ratio (INR), liver enzymes: ALT, AST and alkaline phosphatase (ALP), creatinine, and complete blood count (RBC, Hct, Hgb, MCV, WBC and platelets).

 All the laboratory tests were collected only at admission, except for ALT, AST, ALP, INR, and CRP, which were also collected during hospitalization, including their peak values, and eventually at discharge.

 In our study, the main outcome was defined as a type of oxygen delivery device, need for ICU admission, duration of hospital stay, in-hospital death, or complete recovery.

 Furthermore, we used the collected data to divide the in-hospital disease severity into mild, moderate, and severe according to NIH’s (National Institution of Health’s) COVID-19 Treatment Guidelines 2022 (accessed in January 2023) (Table 1).^14^

**Table 1:** Inhospital disease prognosis and their definitions

**Disease severity **	**Definition**
Mild	Fever and upper respiratory tract infection
Moderate	Shortness of breath, a respiratory rate more than 30 per minute in resting state, oxygen saturation less than or equal to 93%; or partial pressure of arterial oxygen (PaO2)/oxygen concentration (FiO2) ≤ 30 mm Hg (1 mm Hg = 0.133 kPa)
Severe	Respiratory failure requiring mechanical ventilation, shock, or other organ failure requiring ICU monitoring and treatment

 To identify specific clinical and laboratory features in patients with abnormal liver enzymes, patients were categorized into three groups according to their initial ALT levels: one time above the upper limit of normal (ULN), twice above the ULN, and ≥ 3 times above the ULN.

###  Sampling

 The sample size was calculated using the sample size calculator Raosoft®; using 5% as the margin of error and 95% confidence level, with 50% response distribution, and a predicted population size of 4233, which is the number of COVID-19 admissions to Jordanian Hospitals in March 2021. This gave the recommended sample size of approximately 353.

###  Statistical Analysis 

 All statistical analysis was done using IBM SPSS Statistics® version 25. A *P* value < 0.05 was considered significant. Categorical variables were presented as frequencies, and comparisons between groups were performed using a chi-squared test.

 Continuous variables were presented as means and standard deviations (SD). One-way analysis of variance (ANOVA) was performed to compare findings between more than two groups. Pearson’s correlation coefficient was used to measure the linear correlation.

## Results

 The total number of included patients was 359. Most patients were from the middle region of Jordan, with ages ranging between 45 and 74 years, and most were female (Table 2). Almost all the patients’ disease severity was moderate. The vast majority presented with shortness of breath, and only a few presented with gastrointestinal symptoms, as shown in Table 3. The most prevalent comorbidities among the patients were hypertension and type 2 diabetes mellitus. However, many other conditions were also present, including gastrointestinal, lung, autoimmune, and heart diseases, as shown particularly in Table 4. All of those patients have fully recovered and were discharged. 

**Table 2:** Demographic data of 359 patients hospitalized for COVID-19

**Characteristics**	**No. (%) **
Region	
Middle	285 (79.6%)
North	44 (12.3%)
South	29 (8.10%)
Age ranges	
(18-24)	2 (0.56%)
(25-34)	24 (6.69%)
(35-44)	38 (10.6%)
(45-54)	82 (22.8%)
(55-64)	83 (23.1%)
(65-74)	88 (24.5%)
(75 + )	42 (11.7%)
Sex	
Female	197 (54.9%)
Male	162 (45.1%)

**Table 3:** Disease severity and presenting symptoms of 359 patients hospitalized for COVID-19

**Characteristics**	**No. (%) **
Disease severity	
Mild	26 (7.24%)
Moderate	298 (83.0%)
Severe	35 (9.75%)
Presenting symptoms	
Shortness of breath	254 (73.6%)
Cough	147 (42.6%)
Chest pain	28 (8.12%)
Positive PCR	36 (10.4%)
General weakness/fatigue	75 (21.7%)
Fever	78 (22.6%)
Chills	5 (1.45%)
Headache	15 (4.35%)
Dizziness	6 (1.74%)
Loss of appetite	5 (1.45%)
Arthralgia	7 (2.03%)
Abdominal pain	6 (1.74%)
Nausea/vomiting	15 (4.41%)
Diarrhea	20 (5.88%)

**Table 4:** Comorbidity of 359 patients hospitalized for COVID-19

**Comorbidities**	**No. (%) **
DM	
Type 2 DM	183 (51.3%)
Type 1 DM	4 (1.12%)
Hypertension	218 (60.9%)
IHD	41 (11.4%)
Heart failure	25 (6.96%)
Dyslipidemia or hyperlipidemia	27 (7.52%)
CKD	24 (6.69%)
Other heart diseases (cardiomegaly, valvular disease, AF)	18 (5.01%)
GI diseases (IBS, GERD)	17 (4.74%)
Asthma	29 (8.08%)
COPD	8 (2.23%)
Malignancies	6 (1.67%)
Thyroid diseases	31 (8.64%)
Autoimmune conditions*	25 (6.96%)
Blood diseases	10 (2.79%)
CVA	4 (1.11%)
Other lung diseases	5 (1.39%)

DM, Diabetes mellitus; IHD, Ischemic heart disease; CKD, Chronic kidney disease; AF, Atrial fibrillation; GI, Gastrointestinal; IBS, Irritable bowel syndrome; GERD, Gastroesophageal reflux disease; COPD, Chronic obstructive pulmonary disease; CVA, Cerebrovascular accident. *Autoimmune conditions, including psoriasis, sarcoidosis, systemic lupus erythematosus, Sjogren’s, multiple sclerosis, ulcerative colitis, and rheumatoid arthritis.

 Approximately 39.55% of the patients had elevated liver enzymes, and 5.85% were elevated more than twice the ULN. Among these patients, 40.8% had both AST and ALT elevation, 52.1% had AST elevation alone, and 7.0% had ALT elevation alone.

 Most patients with normal liver enzymes had moderate disease severity (82.9%), with (7.83%) needing ICU admission. There was no significant correlation between disease severity and the need for ICU admission between the four groups, as shown in Table 5.

**Table 5:** Comparison of the disease severity and outcome (need for ICU admission) according to the degree of liver enzyme elevation, separated by the elevation level

	** Normal **	** Elevated one time (1.01-1.99x ULN)**	** Elevated two times (2.00-2.99x ULN) **	** Elevated more than three times (≥3x ULN) **	***P*** ** overall**
Disease severity					0.724
Mild	14 (6.45%)	11 (9.09%)	1 (6.25%)	0 (0.00%)	
Moderate	180 (82.9%)	101 (83.5%)	13 (81.2%)	4 (80.0%)	
Severe	23 (10.6%)	9 (7.44%)	2 (12.5%)	1 (20.0%)	
Disease outcome					
ICU admission	17 (7.83%)	9 (7.44%)	1 (6.25%)	0 (0.00%)	0.890

 There were significant differences between patients with abnormal and normal liver enzymes regarding age (*P* = 0.023) and sex (*P* = 0.018), with most patients with elevated liver enzymes being older males. Finally, regarding the comorbidities of patients, there was no difference between the four groups except for comorbid autoimmune conditions (including psoriasis, sarcoidosis, systemic lupus erythematosus, Sjogren’s, multiple sclerosis, ulcerative colitis, rheumatoid arthritis) with a *P* value of 0.020.

 The peak and at-discharge values of AST and ALT showed significant differences between the four groups (*P* < 0.001). The CRP, ALP, BIL, INR, and D-dimer values showed no significant differences between the four groups. Regarding the disease outcomes, there was no significant difference in the need for ICU admission nor oxygen delivery devices, except for the length of stay (*P* = 0.003), as shown in Table 6.

**Table 6:** Comparison of the frequency, clinical characteristics, and comorbidities of patients with COVID-19 according to the degree of liver enzyme elevation, separated by the level of elevation

**Characteristics**	**Normal ** **(n=217)**	** Elevated one time (1.01-1.99x ULN)** **(n=121)**	** Elevated two times (2.00-2.99x ULN) (n=16)**	** Elevated more than three times (≥3x ULN) (n=5)**	***P*** ** overall**
Laboratory results					
Peak ALT	29.3 (25.9)	47.1 (31.1)	90.3 (27.8)	492 (72.8)	**<0.001 **
Peak AST	29.8 (18.5)	50.0 (25.5)	110 (146)	98.7 (94.4)	**<0.001 **
ALT at discharge	29.2 (29.3)	41.4 (27.7)	78.8 (38.8)	84.0 (36.8)	**<0.001 **
AST at discharge	24.8 (16.2)	30.9 (14.7)	44.8 (18.4)	34.4 (25.4)	**<0.001 **
CRP with peak readings of ALT/AST	98.7 (81.2)	127 (71.7)	88.1 (58.8)	86.6 (63.6.)	0.296
ALP	78.5 (27.5)	94.1 (71.2)	108 (80.2)	68.0 (50.9)	0.191
Total bilirubin	8.05 (4.95)	17.8 (61.1)	9.91 (3.13)	9.35 (2.27)	0.258
INR	1.09 (0.39)	1.07 (0.28)	1.18 (0.49)	0.94 (0.55)	0.563
D-Dimer	3.34 (19.9)	1.60 (2.35)	1.47 (1.26)	0.50 (1.7)	0.875
Disease outcome					
ICU length of stay	11.1 (8.43)	24.4 (15.1)	8.00 (.)	0 (0)	0.065
Oxygen delivery device					
NRM	0.39 (0.49)	0.42 (0.50)	0.56 (0.51)	0.40 (0.55)	0.562
SFM	0.53 (0.50)	0.60 (0.49)	0.50 (0.52)	0.40 (0.55)	0.537
NC	0.12 (0.33)	0.16 (0.37)	0.19 (0.40)	0.40 (0.55)	0.247
BiPAP	0.02 (0.13)	0.02 (0.13)	0.06 (0.25)	0.00 (0.00)	0.636
CPAP	0.06 (0.23)	0.02 (0.16)	0.06 (0.25)	0.20 (0.45)	0.229
Length of stay	8.04 (7.58)	10.0 (9.33)	10.6 (12.8)	21.0 (18.1)	** 0.003 **

Data were presented as means (SD). Analysis was done using the ANOVA test. *P* value < 0.05 is considered significant. NRM; non-rebreather mask, SFM; simple face mask, NC; nasal cannula, BiPAP; bi-level positive airway pressure, CPAP; continuous positive airway pressure.

 The four groups had no significant difference in the presenting symptoms, disease severity, comorbidities, and laboratory tests (WBC, RBC, Hct, Hgb, MCV, creatinine).

 According to the yielded Pearson’s coefficients, there was no significant association between elevated liver enzymes and CRP or D-Dimer.

## Discussion

 In this multicenter retrospective cohort study involving 359 hospital inpatients infected with COVID-19, we investigate the changes to the liver enzymes and their association with prognosis and disease outcome. Our analysis revealed that the most common form of AST alteration across all three stages of disease severity was 1.01-1.99x ULN, and most (83%) patients had moderate disease severity.

 Earlier studies have similarly found that severe patients, upon admission, show a greater frequency of AST increase compared with ALT.^15,16^ Other studies also report that AST levels differ the most between severe and non-severe groups.^17^ This contradicts other studies that found that ALT levels were significantly higher than AST levels in patients with severe COVID-19.^18,19^

 The current study is in line with other studies that conclude that liver enzyme elevation is significantly higher in male patients than female patients,^20-22^ with the average age being 55.97 ± 13.12 years.^22^ However, other studies show no significant difference between sexes^7^ or age.^7,20^

 As for the comorbidities, a study shows no significant correlation between elevated liver enzymes and any pre-existing comorbidities,^20^ whereas another study shows that patients with severe disease compared with those with non-severe disease more commonly have pre-existing comorbidities (38.7% and 21.0%, respectively).^15^ However, our study identified a significant association between autoimmune diseases and elevated liver enzymes (*P* = 0.02). Notably, 6.96% of the patients presented with an autoimmune disease.

 Previous studies show that total BIL levels are higher in severe disease states,^7,17,23^ are associated with an increased mortality risk,^17^ and have an increased need for vasopressor therapy and mechanical ventilation in patients with COVID-19.^24^ In our study, the levels of total BIL correlate with the groups of elevated liver enzymes, but no significant difference in the means is found (*P* = 0.258).

 A study in China shows that elevated liver enzymes are associated with cough as an initial COVID-19 symptom,^6^ and in another study from Iraq, liver enzyme elevations are more common in symptomatic than asymptomatic patients.^10^ In our analysis, there was no significant difference in the presenting symptoms of the patients with normal or abnormal liver enzymes or between the three different groups of elevated liver enzymes.

 A study shows that INR has no independent association with severe COVID-19 mechanical ventilation,^23^ whereas in another study, INR significantly differs between severe and critical groups.^7^ Our analysis showed no significant difference between the normal or abnormal groups or between the three groups of elevated liver enzymes and the degree of INR elevation.

 In one study, serum albumin was lower in COVID-19 patients than in healthy controls, but there was no difference between severe and non-severe groups.^16^ However, the serum level of albumin was not routinely measured in the Jordanian hospitals during the period of our study; therefore, it was not assessed.

 The current study showed no significant differences between the groups regarding the need for ICU admission and oxygen delivery devices. On the other hand, the length of stay varied significantly (*P* = 0.003), with the highest mean of 21 days observed among patients with liver enzyme elevation of three times or more the ULN, which is in line with other studies that suggested that patients with abnormal liver enzymes during the course of the disease had a longer hospital stay and more severe symptoms.^25,26^ Nevertheless, a prospective study from Iran showed that patients with ALT and AST levels of more than 40 IU/L and liver injury on admission had significantly greater odds of death, ICU admission, and requirements.^20^

 Numerous studies have investigated the clinical implications of liver enzymes in patients with SARS-CoV-2 infection, with varying results.^27-30^ These studies have classified COVID-19-related liver injury into two categories, cholestatic and hepatocellular, based on the significance of the liver enzymes observed.^27-30^ For instance, a study conducted in China revealed that patients with severe and critical COVID-19 had higher levels of gamma-glutamyl transferase (GGT) than those with moderate illness, which may indicate cholangiocyte injury.^31^ Our study supports the hepatocellular type of injury as elevated liver transaminases show significant abnormality. However, we could not fully assess the cholestatic pattern of liver injury because serum levels of GGT are not routinely tested in most centers where we based our study. Meanwhile, Davidov-Derevynko et al showed that liver aminotransferase elevation is more predominant than ALP increment in their sample of 382 patients, suggesting a hepatocellular pattern of liver damage as opposed to a cholestatic one.^28^ Although angiotensin-converting enzyme 2 (ACE-2) is highly expressed in bile duct cells, which indicates a potential for liver injury due to cholangiocyte dysfunction in patients with COVID-19,^32^ our study found no significant difference in ALP levels among the three groups, with a *P* value of 0.191.

 Several limitations should be considered when interpreting the results of this study: not taking into consideration the impact of drug history and medications given during the hospital stay; the effect of secondary bacterial infection on the patient, or the association between shock or hypoxia, which are often observed in critically ill patients with COVID-19, and acute hepatitis. Poor documentation led to several unknown factors that could influence liver enzyme levels, such as patient BMI and average alcohol intake, and many other blood tests that were not routinely assessed, could not be studied.

 Furthermore, due to the retrospective nature of the study, it was amenable to missing values of variables that were not among our inclusion criteria (i.e. other than ALT and AST), for which the analysis was based on the values present.

 The strengths of this study include a relatively large sample size, including data from multiple centers from different regions across Jordan, covering a major peak of a COVID-19 wave in Jordan, excluding patients who were pregnant or with pre-existing liver disease (those with previous documentation of liver disease or hepatitis B and C) and studying a wide range of variables in association with the liver aminotransaminases.

## Conclusion

 Our study concludes that liver enzyme abnormality is common among COVID-19 hospital inpatients, with the most common abnormality being AST elevation. They are more common in patients with pre-existing autoimmune diseases and in older males. Liver enzyme abnormalities are associated with a longer hospital stay.
